# Routes of Delivery for CpG and Anti-CD137 for the Treatment of Orthotopic Kidney Tumors in Mice

**DOI:** 10.1371/journal.pone.0095847

**Published:** 2014-05-02

**Authors:** Jennifer A. Westwood, Titaina C. U. Potdevin Hunnam, Hollie J. Pegram, Rodney J. Hicks, Phillip K. Darcy, Michael H. Kershaw

**Affiliations:** 1 Division of Cancer Research, Peter MacCallum Cancer Centre, St Andrew's Place, East Melbourne, Australia; 2 Centre for Cancer Imaging, Peter MacCallum Cancer Centre, St Andrew's Place, East Melbourne, Australia; 3 Sir Peter MacCallum Department of Oncology, University of Melbourne, Parkville, Australia; 4 Department of Immunology, Monash University, Prahran, Australia; Istituto Superiore di Sanità, Italy

## Abstract

We have found previously that the tumor cell lines, Renca (a renal cancer) and MC38 (a colon tumor) which had been injected subcutaneously in mice, could be successfully treated with a combination therapy of an oligodeoxynucleotide (CpG1826) (injected intratumorally) and anti-CD137 antibody (injected intraperitoneally). Thus the combination treatment was expected to initiate a “danger” signal via TLR9 on immune cells, and the anti-CD137 was expected to further activate T cells. In the present study, we found that several other tumor types injected subcutaneously could also be successfully treated with this combination therapy. In addition, we wished to determine if the treatment could work as effectively in an orthotopic metastatic model, which is more physiologically relevant to cancer in humans. Renca was selected as we were familiar with injecting this orthotopically into the outer cortex of the kidney in mice, and it spontaneously metastasizes to lung and abdominal sites. We tested various routes of delivery of CpG combined with intraperitoneal delivery of anti-CD137. Orthotopic tumors were injected with CpG intratumorally, using ultrasound-guided delivery on multiple occasions, combined with anti-CD137 intraperitoneally. A reduction in primary tumor size was observed following intratumoral injection of CpG compared to other treatments. We found that there was a statistically significant increase in survival of mice with orthotopic Renca tumor following intratumoral injection of CpG. However, we determined that the most effective route of delivery of CpG was intravenous, which led to further significantly enhanced survival of mice when combined with anti-CD137 intraperitoneally, likely due to inhibition of metastatic disease. Our data supports future development of this combination therapy for cancer.

## Introduction

CpGs are unmethylated oligodeoxynucleotides which mimic bacterial DNA. They bind to Toll-like receptor 9 (TLR9), which is expressed on murine monocytes, macrophages, plasmacytoid dendritic cells (DCs) and B cells. Ligation of TLR9 by CpG can initiate an inflammatory or “danger” signal from these cells, enabling immune cell recruitment and antigen presentation (reviewed in [1] and [2]). Anti-CD137 (αCD137) is an agonist antibody specific for CD137 expressed on activated T cells (more so on CD8+ than CD4+ T cells), DCs, natural killer cells (NKs), granulocytes and endothelial cells at inflammation sites. Ligation of CD137 on T cells leads to enhanced T cell proliferation, T cell cytotoxicity, prevents activation-induced cell death and prevents immune tolerance (reviewed in [3] and [4]). In a previous study we found that combined treatment of CpG1826 and αCD137 promoted tumor regression and increased survival in subcutaneous (SC) tumor models using MC38 (colon carcinoma) and Renca (renal cell carcinoma) in mice [5].

In the present study we expanded the testing of this therapy to further SC models: B16F10 (melanoma) and CT26 (colon carcinoma), finding CpG alone or in combination with αCD137 increased therapeutic efficacy. As it is increasingly clear in the literature [6]–[9] that orthotopic models give a more physiologically relevant indication of the efficacy of treatments both in mice and humans, we elected Renca to be injected orthotopically into the kidney of mice and treated with combined CpG1826 and αCD137. This produces not only primary renal tumors, but also metastases to other organs such as the lung and liver, which play a major role in disease progression and mortality. This model recapitulates sites of metastasis in human renal carcinoma [10]. Previously we found that orthotopic Renca tumor was minimally responsive to treatment with a combination of three antibodies (anti-DR5, anti-CD40 and anti-CD137) [11] injected intraperitoneally, and therefore we wished to determine if a different immunotherapy (combining CpG and anti-CD137) could have a better impact on metastatic orthotopic kidney cancer.

Initially, we tested SC injections (into the skin) of CpG1826 combined with IP αCD137. Subsequently, we used ultrasound-guided injection of CpG directly IT into kidney tumors. Ultrasound-guided injection of other therapies has been used for other tumors in mice, including those of bladder [12], prostate [13] and liver [14], but not to our knowledge has it been used to treat kidney tumors in mice. We also tested CpG injected intravenously combined with IP αCD137, and found this gave the greatest efficacy of the treatment against metastatic orthotopic kidney cancer.

## Materials and Methods

### Cell lines

The mouse renal cell carcinoma tumor cell line, Renca [15], the melanoma cell line B16F10 (ATCC, Manassas, VA, USA), and the colon tumor lines CT26 (ATCC) and MC38 (ATCC) were maintained in complete medium consisting of RPMI (Gibco, Life Technologies, Grand Island, NY) with 10% heat-inactivated fetal calf serum (MultiSer, Thermo Trace, Melbourne) and additives (2 mM glutamine (Gibco), 100 µg/ml streptomycin (Sigma-Aldrich, St Louis, MO) and 100 U/ml penicillin (Sigma-Aldrich) in a humidified incubator at 37°C with 5% CO_2_.

### Mouse tumor models

Ethics statement: This study was carried out in strict accordance with the recommendations of the Victorian Bureau of Animal Welfare, Department of Primary Industries, and the National Health and Medical Research Council's Australian code of practice for the care and use of animals for scientific purposes. The protocol was approved by the Peter MacCallum Cancer Centre Animal Experimentation Ethics Committee under Permit numbers E1274 and E1328. All efforts were made to minimize suffering. The individual shown in **Figure S1** in this manuscript is the operator of the ultrasound equipment and has given written informed consent (as outlined in PLOS consent form) to appear in this figure.

Wild type female BALB/c and C57BL/6 mice were purchased from the Walter and Eliza Hall Institute of Medical Research (Bundoora, Australia). At 8–12 weeks of age, mice were injected subcutaneously (SC) with either 5×10^5^ B16F10, 1×10^6^ MC38, 5×10^5^ Renca or 1×10^6^ CT26 cells in 100 µl saline. When tumors averaged 48 mm^2^ (for B16F10), 33–35 mm^2^ (for MC38), 38 mm^2^ (for Renca) and 20–33 mm^2^ (for CT26) in size (day 7 for B16F10, day 10 for MC38, day 9 for Renca and day 6–7 for CT26), mice were randomized and assigned into groups of comparable ranges of tumor sizes, with 5–9 mice/group, and treated as described below. Tumors were measured (longest and shortest dimensions) every 2–3 days and tumor area calculated. Mice were culled when tumors reached 200 mm^2^ in size or at the first signs of stress.

For intrakidney tumor experiments, BALB/c female mice were injected with 1×10^5^ Renca cells in 10 µl PBS intrakidney (IK) into the outer cortex of the kidney. On day 12 after tumor injection, mice were randomly assigned into 3 groups: not treated, treated with 10 µl PBS IT plus αCD137 antibody intraperitoneally (IP) or treated with CpG1826 (CpG) IT plus αCD137 antibody IP, as detailed below. As negative controls, some groups received isotype control rat IgG2a (MAC4, produced in-house from European Collection of Cell Cultures hybridoma) instead of anti-CD137 antibody, and/or saline instead of CpG.

### Treatment

Treatment consisted of injections with CpG (CpG1826 = 5′-TCCATGACGTTCCTGACGTT-3 from Coley Pharmaceuticals, Canada) at the dose of 50 µg/50 µl for IT injection of SC tumors, 50 µg/10 µl for IT injection of IK tumors, or 50 µg/200 µl for IV injection, starting on the first day of treatment specified above and then repeated every 2–3 days for a total of 2–4 injections. αCD137 antibody (in-house generated using caprylic acid precipitation) was injected IP, starting on the first day of treatment, at the dose of 100 µg/200 µl. αCD137 injections were repeated every 3–4 days for a total of 3–6 injections. Isotype control antibody (MAC4) was given at the same dose and rate as αCD137 to appropriate control groups of mice.

The method of injecting IK tumors intratumorally is described as follows. Imaging of the kidney tumor was performed using a 40 MHz single-element mechanical transducer with focal length 6 mm; axial resolution 40 µm; lateral resolution 80 µm (Vevo 770 high-resolution small-animal ultrasound system, Visualsonics, Toronto, Ontario, Canada). The fur of the mice was locally shaved over the kidney area then further removed using a depilatory cream (Veet, Reckitt Benckiser Group, Slough, Berkshire, England). Mice were then injected with ketamine hydrochloride (80 mg/kg, Ketalar, Hospira Australia, Mulgrave, VIC, Australia) plus xylazine hydrochloride (10 mg/kg, Ilium xylazil-20, Troy Laboratories, Smithfield, NSW, Australia) and once anesthetized, placed on a temperature-controlled stage of the integrated rail system (Visualsonics). This setting allowed adjustment of the mouse's positioning while maintaining the 30 gauge injection needle mounted on a Hamilton syringe (Hamilton Company, Reno, Nevada, USA) within the transducer-imaging plane. The therapeutic was then delivered to the tumor by moving the tumor into the scan plane using the XYZ controls on the mouse stage.

### Cell depletions

In one experiment, mice were depleted of CD8^+^ T cells, by IP injection of anti-CD8 monoclonal antibody (clone 53-6-72, produced in-house from hybridoma) at the dose of 200 µg/200 µL PBS on day −1 of tumor injection, then 100 µg/200 µL PBS on days 0 and 1, and every 3 to 4 days thereafter after start of treatment. Control monoclonal antibody (MAC4), was given to the control group at the same dose and rate. NK cells were depleted in another group by injection of anti-asialo GM1 (rabbit immunoglobulin, Wako Pure Chemical Industries Ltd, Osaka, Japan): 1 mL was diluted 1/10 in PBS and 200 µL injected on the day of start of treatment, then every 3 to 4 days thereafter.

### Statistical analysis

Statistical significance in the experiments compared survival of mice between the various groups and was determined by the Wilcoxon log-rank (Peto-Prentice) test, using StatsDirect software (version 3.0.86 beta, StatsDirect Ltd, Altrincham, Cheshire, UK). Statistical significance comparing tumor sizes between groups was determined using the Mann-Whitney test and the unpaired T test, using StatsDirect software. Statistical analysis of lung metastases burden and ascites incidence was determined using Fishers' Exact Test, with StatsDirect software.

## Results

### Treatment with CpG alone, or in combination with αCD137, enhanced the survival of mice bearing various subcutaneous tumors

**Figure 1:**
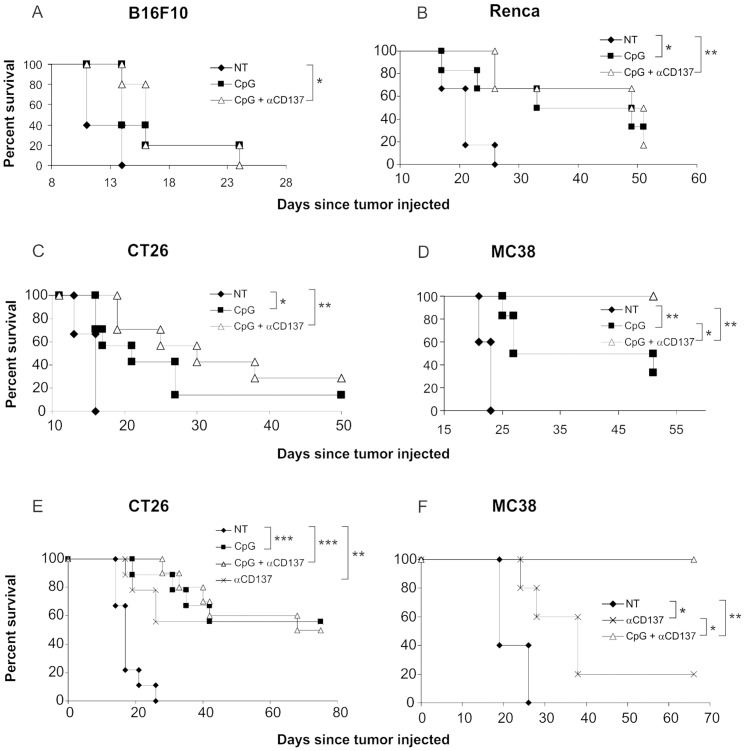
Administration of CpG IT and αCD137 IP significantly enhances survival of mice injected with a variety of SC tumors. Mice were injected subcutaneously with single cell suspensions of B16F10 (A, n = 5), Renca (B, n = 5–6), CT26 (C, n = 6–7, and E, n = 9–10) and MC38 (D, n = 6, and F, n = 5). When tumors reached an average of 20–50 mm^2^ in size, mice were injected with CpG IT (every 2–3 days for 4 doses) and αCD137 IP (every 3–4 days for 3 doses), as outlined in Methods, and survival of mice was monitored. * p<0.05, ** p<0.005, *** p<0.0005.

C57BL/6 mice injected SC with either B16F10 or MC38 tumor cells, and BALB/c mice injected SC with either Renca or CT26 were treated with a combination of CpG IT and αCD137 IP. **Figure 1** **A–F** shows that the combination therapy had a statistically significant effect on the survival in all tumor models in comparison with no treatment (for B16F10: P = 0.0094 (**Figure 1A**), for Renca: P = 0.002 (**Figure 1B**), for CT26: P = 0.0008 (**Figure 1C**) and <0.0001 (**Figure 1E**), for MC38: P = 0.002 (**Figure 1D**) and 0.0039 (**Figure 1F**)). In the Renca, CT26 and MC38 models, injection of CpG alone was also able to increase the survival of mice compared to non-treated mice (**Figure 1B, C, D, E**). In addition, administration of αCD137 alone was also able to increase mouse survival of mice bearing CT26 or MC38 tumors (**Figure 1E and F**). Except for the MC38 model (**Figure 1D and F**), statistical significance was not quite achieved for groups receiving combined therapy with CpG+αCD137 compared to monotherapy with CpG or αCD137 alone. However, given the trend of mice to survive longer after combined treatment in the current study, and the statistically significant improvement in survival afforded by combination therapy in our previous studies [5], we performed the remainder of the current study in Renca using the combination of CpG+αCD137. However, in the experiment shown in **Figure 1B** using the Renca model, we cannot claim that the combined therapy with CpG and αCD137 was more effective than monotherapy with CpG or αCD137 alone. Nevertheless, the use of the combination therapy allowed us to determine the feasibility of treating Renca using the model and schedules described below.

All these tumors were injected SC, but only B16F10 originated from cancer of this organ (melanoma). Both MC38 and CT26 are of bowel cancer origin and Renca is of renal cancer origin. Therefore we wished to determine if the significant survival seen with all these tumors injected SC would apply to the same tumors located orthotopically, a more realistic test of therapy efficacy. As we were familiar with Renca injected into the kidney producing renal cell cancer in mice, we decided to proceed with this model.

### Administration of CpG by various routes in combination with αCD137 IP enhances the survival of mice bearing orthotopic Renca tumors

**Figure 2:**
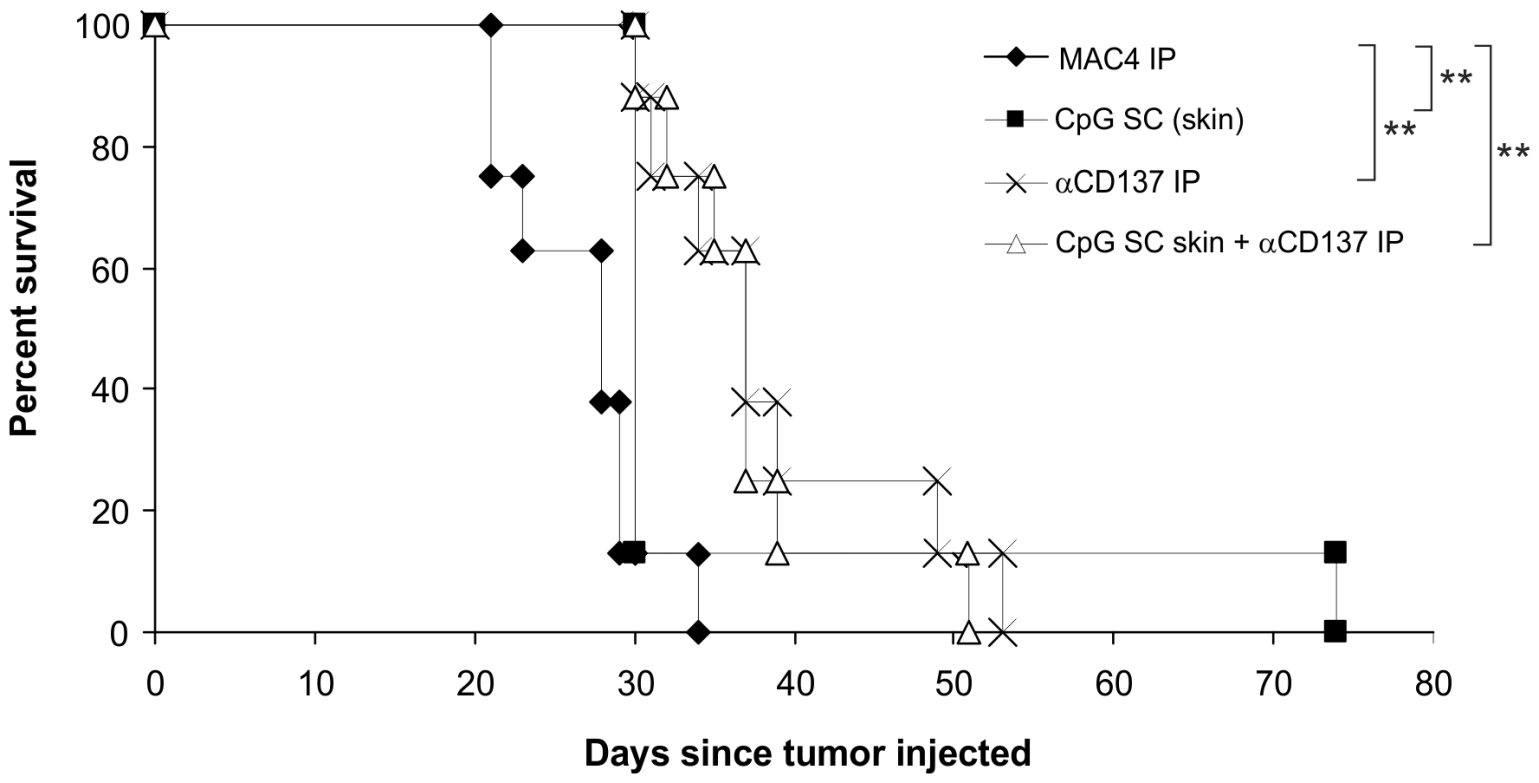
SC CpG and IP αCD137 treatment of kidney tumor-bearing mice does not lead to tumor free mice. Renca cells were injected orthotopically into the outer cortex of the kidney, and then mice were treated starting on day 11 with either isotype control antibody MAC4 IP or αCD137 IP (every 3–4 days for 6 injections), and/or CpG injected SC into the skin (every 2–3 days for 4 injections) (n = 8 per group). ** p<0.005.

Renca was injected orthotopically into the outer cortex of the kidney. Orthotopic injection of Renca produces a renal tumor, which frequently metastasizes to the lung and occasionally to the liver, diaphragm and peritoneum. Orthotopic Renca is thus a very much more difficult mouse model in which to achieve survival with therapies compared with Renca SC which does not metastasize at all. In previous mouse studies and human clinical trials by other investigators, CpG has been used as a monotherapy or combined with other therapies for treating solid and hematological cancers (including melanoma, renal cell cancer, breast cancer and lymphomas), and has been injected by several routes, including SC, IT and intravenously (IV) [16]–[20]. In some of these studies intratumoral injection of CpG alone has been sufficient to induce regression or slow the growth of tumors. Given this, we initially tried injecting CpG into the skin (SC) with IP αCD137 to determine if this would affect the survival of the mice. **Figure 2** shows that this led to no long term survivors, either as single therapeutic agents, or in combination, although all were statistically significant compared with not treated mice (P = 0.0038 for CpG only, P = 0.0008 for αCD137 only and P = 0.0006 for combined treatment).

Intratumoral delivery of CpG into SC tumors has been previously demonstrated to be more effective than systemic or remote injection of CpG in some cases [16], [21], and given the success of IT injection in SC tumors (**Figure 1**) we wished to determine if this could be replicated in the orthotopic metastatic tumor model. We therefore tested injecting the CpG intratumorally into kidney tumors combined with IP αCD137 to determine if this treatment regimen could induce an enhanced degree of mouse survival similar to that observed with subcutaneous Renca tumors. The therapeutic injection of kidney tumors was ultrasound-guided as per the method described above. **Figure S1A and B** depict the set-up of mouse, syringe and needle and ultrasound imaging equipment. To validate the accuracy of ultrasound-guided IT injection **Figure S1C** shows a kidney tumor filled with a dye (Trypan blue) which was performed during development of this technique. In experiments where CpG was injected IT, it was possible to see the solution being injected into the tumor, as shown in the montage of time-lapse photography (**Figure S2** and **Movie S1**). Permeation of tumors by CpG solution was evident by an observed reduction in density of the tumor during injection of the solution. Injection of material into each renal tumor was displayed on-screen in all ultrasound guided experiments, and permeation of CpG into tumors was readily and reproducibly observed.

**Figure 3:**
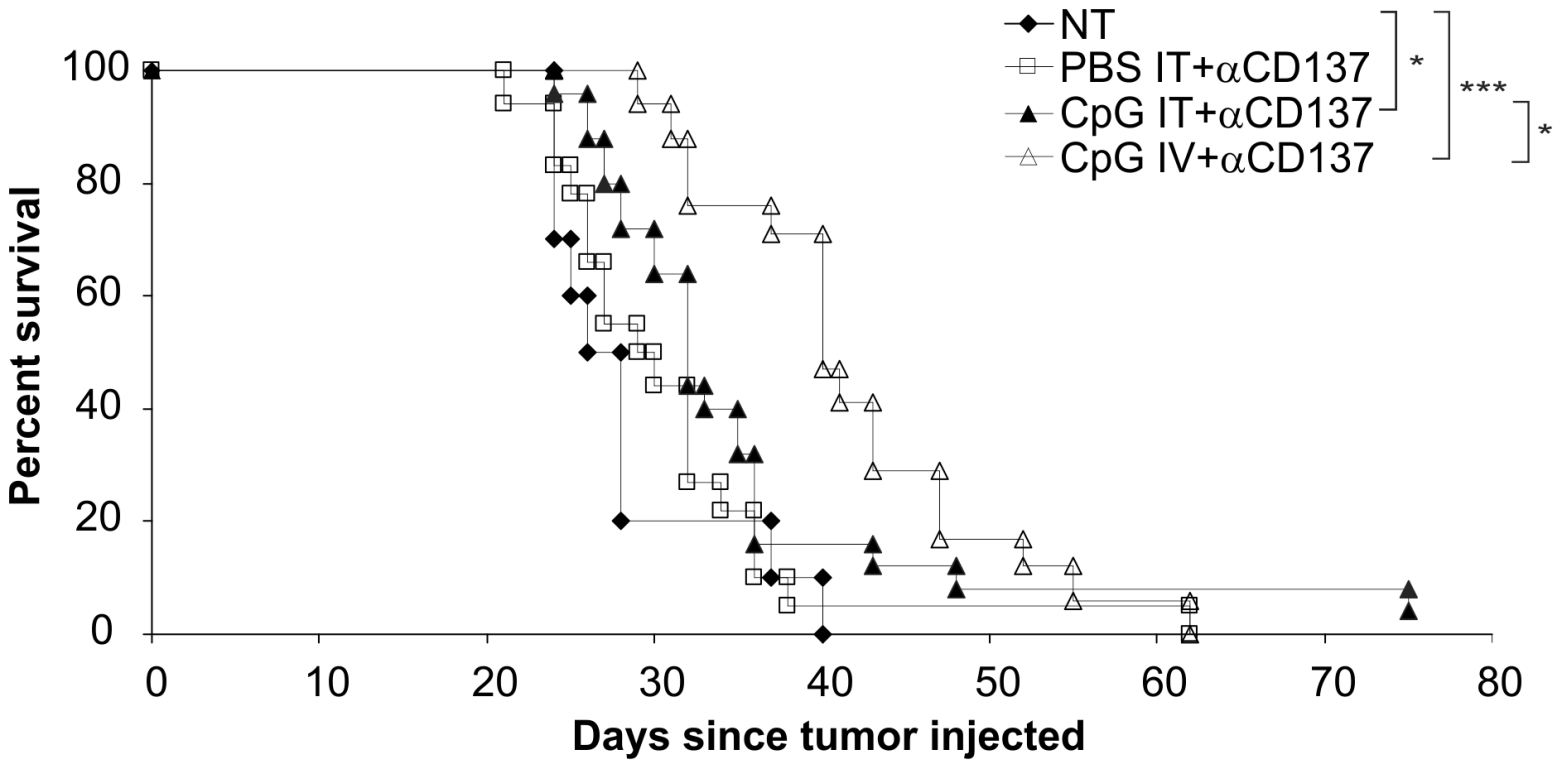
Mice bearing kidney tumors survive longer when CpG is injected IV compared to IT injection. Renca was injected orthotopically into the outer cortex of the kidney, and then mice were treated with either CpG or PBS injected IT using ultrasound imaging assistance on days 12, 14, 17 and 19, or with CpG IV on days 12, 14 and 17, in combination with αCD137 IP (on days 12, 15 and 19) for all three groups (pooled results of 2 different experiments, n = 10 in not treated (NT) group, and n = 17–25 in treated groups). * p<0.05, *** p<0.0005.

**Figure 3** shows the pooled results of two experiments in which mice bearing kidney tumors were treated with CpG IT, performed using this methodology, in combination with αCD137 IP. For direct comparison, a group was treated with CpG IV plus αCD137 IP within the same experiment. In **Figure 3**, three groups were treated, either with PBS IT plus αCD137 IP or CpG IT plus αCD137 IP or CpG IV plus αCD137, and compared with a not treated group. The data for this figure was derived from two separate experiments, which are shown individually in **Figure S3**. The majority of mice which had to be culled in all groups had large kidney tumors, but mice were also culled because of ascites in the peritoneum, or due to large numbers of tumor metastases in the lungs, or large abdominal tumors (**Table 1**). There was a significant increase in survival of both CpG groups (P = 0.0138 for IT injected group, and P = <0.0001 for the IV injected group, **Figure 3**) compared with the no treatment group. In addition, there was a statistical significant difference between these two treatment groups (P = 0.0057) with the CpG IV treated group surviving longer than the CpG IT treated group. There was no significant difference in survival with αCD137 alone group compared with the not treated group (P = 0.3654).

**Table 1:** Summary of necropsies performed on individual mice on day of death or at the end of experiment for the two experiments shown in Figure S3.

Experiment 1						Experiment 2					
Group	mouse #	primary kidney tumor mm2	lung met estimate	ascites score	# of other mets (liver, abdominal or diaphragm)	Group	mouse #	primary kidney tumor mm2	lung met estimate	ascites score	# of other mets (liver, abdominal or diaphragm)
Not treated	1	165	*	*	*	Not treated	1	242	TNTC	+++	5
	2	154	TNTC	++	3		2	84	TNTC	0	1
	3	387	TNTC	++	4		3	372	TNTC	+	1
	4	232	20	0	1		4	244	TNTC	+	2
	5	48	TNTC	0	1		5	608	10	+	6
	Average	**197±56**					Average	**310±87**			
PBS it +αCD137	6	285	TNTC	+	0	PBS it +αCD137	6	347	11	0	5
	7	147	TNTC	0	2		7	368	5	++	18
	8	596	*	+	16		8	533	10	0	15
	9	576	0	++	0		9	190	TNTC	0	1
	10	388	0	+++	5		10	402	TNTC	0	1
	11	177	TNTC	0	0		11	209	TNTC	0	1
	12	27	TNTC	0	1		12	267	TNTC	+	0
	13	249	TNTC	++	2		13	441	TNTC	++	0
	14	26	TNTC	0	0		14	392	TNTC	+	0
	15	44	TNTC	0	0		15	132	2	+++	1
	Average	**252±67**					Average	**328±40**			
CpG it +αCD137	16	38	*	*	*	CpG it +αCD137	16	96	TNTC	0	0
	17	18	TNTC	0	0		17	230	TNTC	+	1
	18	196	TNTC	0	4		18	*	*	*	*
	19	193	0	0	6		19	465	TNTC	++	0
	20	480	20	+	3		20	283	TNTC	++	0
	21	15	TNTC	0	3		21	138	TNTC	+	2
	22	42	TNTC	0	0		22	306	TNTC	+++	2
	23	281	2	++	6		23	39	TNTC	0	1
	24	17	TNTC	0	0		24	10	TNTC	0	0
	25	412	TNTC	+++	1		25	100	TNTC	0	2
	26	315	TNTC	+++	2		26	623	TNTC	+++	3
	27	0	10	0	4		27	3	TNTC	0	0
	Average	**167±49**					28	0	0	0	0
CpG iv +αCD137	28	177	4	0	0		Average	**191±57**			
	29	355	TNTC	0	7	CpG iv +αCD137	29	253	TNTC	+++	9
	30	370	0	+	2		30	576	TNTC	+++	8
	31	137	*	*	*		31	452	10	+++	4
	32	50	TNTC	+	0		32	181	TNTC	0	0
	33	198	TNTC	+++	4		33	3	TNTC	0	1
	34	384	TNTC	+	0		34	320	TNTC	++	1
	35	370	TNTC	+	2		35	0	TNTC	0	0
	36	463	4	0	0						
	37	750	TNTC	+++	0		Average	**255±82**			
	Average	**325±63**									

Size of primary kidney tumor for each mouse is presented, and the average for each group ± SEM. Degrees of ascites were scored as + (slight), ++ (intermediate), +++(copious). * mouse found decayed or eaten, so no result obtained for this parameter. TNTC, too numerous to count.

Measurements of kidney tumors during ultrasound-guided procedures revealed that by day 19 tumor growth was inhibited significantly with intratumoral injection of CpG combined with intraperitoneal αCD137 when compared to not treated (**Figure S4A**), but was not significantly inhibited when compared with PBS injected intratumorally combined with αCD137 (**Figure S4B,C**). All mice died with large renal tumors and/or metastatic burden. The primary tumor sizes of mice in each group on the day of death were compared within each experiment, shown in **Table 1**, and subjected to statistical analysis (**Table 2**). Using the unpaired single-sided T test, tumor size following IT CpG delivery combined with αCD137 was statistically significantly smaller compared with tumors following IV delivered CpG plus αCD137 in Experiment 1 (P = 0.0296). Analysis of tumor size in Experiment 2 revealed that tumors in mice receiving CpG IT plus αCD137 were significantly smaller than those receiving PBS IT plus αCD137 (P = 0.032 T test). There was no statistically significant difference between primary tumor size at death in mice in other treatment groups. Taken together, these experiments suggest that CpG delivered IT could reduce the size of primary kidney tumors.

**Table 2:** Statistical analysis of primary tumor size.

	Experiment 1		Experiment 2	
	Mann-Whitney	T test unpaired 1-sided	Mann-Whitney	T test unpaired 1-sided
CpG IT+αCD137 vs CpG IV+αCD137	0.059	0.029	0.664	0.267
CpG IT+αCD137 vs NT	0.574	0.348	0.278	0.145
CpG IV+αCD137 vs NT	0.309	0.077	0.755	0.328
CpG IT+αCD137 vs PBS IT+αCD137	0.282	0.163	0.059	0.030
CpG IV+αCD137 vs PBS IT+αCD137	0.435	0.216	0.474	0.194
PBS IT+αCD137 vs NT	0.859	0.304	0.767	0.413

Primary tumors presented in **Table 1** were analyzed for statistically significant differences in sizes between treatment groups using both 2-sided nonparametric Mann-Whitney and by unpaired single-sided parametric T tests. P values are depicted (P>0.05 considered not significant).

The majority of kidney-tumor bearing mice developed lung metastases (**Table 1**), resulting in extensive lung burden at the time of death with the number of metastases often being too numerous to count (TNTC). When the proportion of mice with lung metastases TNTC was compared between mice receiving systemic or intratumoral CpG, no statistically significant difference was observed (**Table 3**). Similarly, when the proportion of mice developing ascites was compared to those without ascites, no statistically significant difference between mice in any treatment group was observed (**Table 4**). In addition, no statistically significant difference was observed in the number of abdominal metastases between mice receiving any treatment (**Table 5**).

**Table 3:** Statistical analysis of lung metastases burden.

	Experiment 1	Experiment 2
CpG IT+αCD137 vs CpG IV+αCD137	1.000	1.000
CpG IT+αCD137 vs NT	1.000	1.000
CpG IV+αCD137 vs NT	1.000	1.000
CpG IT+αCD137 vs PBS IT+αCD137	0.642	0.135
CpG IV+αCD137 vs PBS IT+αCD137	1.000	0.338
PBS IT+αCD137 vs NT	1.000	0.588

Data from **Table 1** comparing groups of mice receiving various treatment regimens were analyzed statistically. A Fisher's exact test was performed on the data, and was based on the proportion of mice with extensive lung metastases (classified as TNTC or too numerous to count) compared to mice with lower lung burden or absence of lung metastases. P values are depicted (P>0.05 considered not significant).

**Table 4:** Statistical analysis of ascites incidence.

	Experiment 1	Experiment 2
CpG IT+αCD137 vs CpG IV+αCD137	0.198	1.000
CpG IT+αCD137 vs NT	1.000	0.338
CpG IV+αCD137 vs NT	0.650	0.576
CpG IT+αCD137 vs PBS IT+αCD137	0.669	1.000
CpG IV+αCD137 vs PBS IT+αCD137	0.650	1.000
PBS IT+αCD137 vs NT	1.000	0.580

Data from **Table 1**, comparing groups of mice receiving various treatment regimens, were analyzed statistically. A Fisher's exact test was performed for the equivalence of ascites based solely on the presence or absence of ascites in the various treatment groups. P values are depicted (P>0.05 considered not significant).

**Table 5:** Statistical analysis of other metastases (liver, abdominal or diaphragm).

	Experiment 1		Experiment 2	
	Mann-Whitney	T test unpaired 1-sided	Mann-Whitney	T test unpaired 1-sided
CpG IT+αCD137 vs CpG IV+αCD137	0.2722	0.1843	0.232	0.0777
CpG IT+αCD137 vs NT	0.9714	0.3788	0.0579	0.0593
CpG IV+αCD137 vs NT	0.3385	0.3359	0.7424	0.4428
CpG IT+αCD137 vs PBS IT+αCD137	0.2925	0.4917	0.3746	0.0786
CpG IV+αCD137 vs PBS IT+αCD137	0.9091	0.3085	0.9457	0.3749
PBS IT+αCD137 vs NT	0.3477	0.4473	0.3823	0.3537

Statistical analysis of data from **Table 1** comparing the number of metastases in sites other than lung in mice receiving various treatments using both 2-sided nonparametric Mann-Whitney and by unpaired single-sided parametric T tests. P values are depicted (P>0.05 considered not significant).

For the combination therapy, comparison of the mice injected SC with CpG in **Figure 2** with the mice injected IT with CpG in **Figure 3** showed no statistical difference (P = 0.0887). CpG injected alone was also not statistically different from the combination therapy with CpG injected IT (P = 0.4563).

Our main aim in this study was to determine the feasibility of ultrasound-guided administration of therapy for kidney tumors. We combined the αCD137 and CpG reagents since this combination has been demonstrated to be effective against some subcutaneous tumors. However, in the present study, we did not determine the contributions of each reagent individually, and it remains possible that there was no contribution to therapy from αCD137 in the kidney tumor setting.

**Figure 4:**
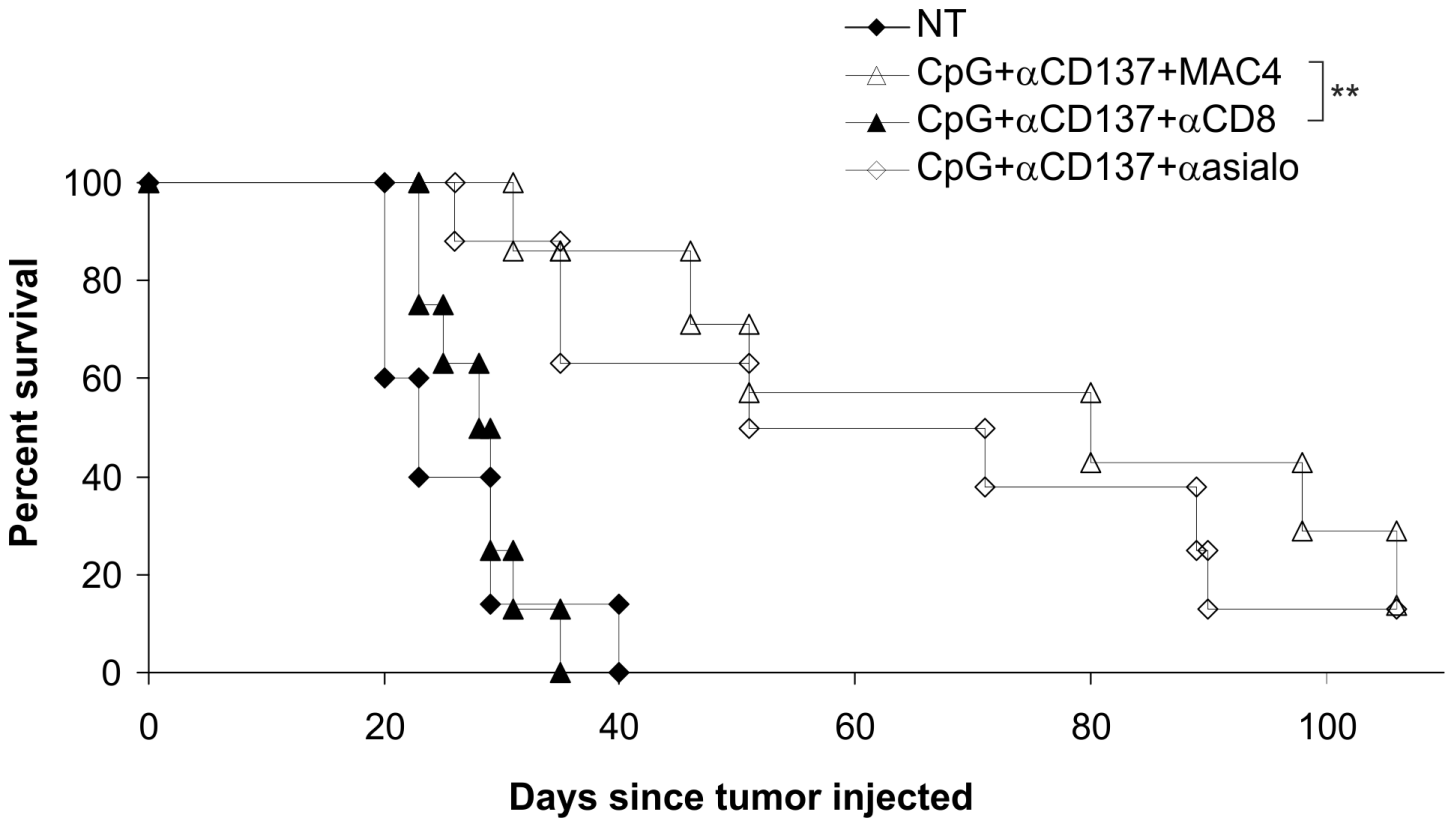
CD8^+^ T cells are essential for enhanced survival of mice bearing kidney tumors and treated with CpG IV and αCD137 IP. CpG was injected three times in combination with αCD137. Injections of CpG (IV) and αCD137 (IP) were given on days 10, 13, and 16 after tumor was injected into the kidney cortex. Depletion of CD8^+^ T cells or NK cells was performed on some groups with αCD8 (IP) or αasialo GM1 (IP) respectively, and another group received MAC4 isotype control antibody (IP) (n = 5–7 per group). ** p<0.005.

To ascertain the mechanism of the enhanced effect of systemic administration of CpG we performed an experiment in which various immune subsets were depleted. Three IV injections of CpG together with IP αCD137 led to significantly longer survival (P = 0.002) of mice compared to not treated mice (**Figure 4**). Previously Uno et al [22] showed that CD8 and IFNγ were necessary for a combination therapy three monoclonal antibodies, including anti-CD137. Thus we explored depletion of either CD8^+^ T cells or NK cells in our model. Results (**Figure 4**) revealed that CD8^+^ T cells were crucial for this effect (P = 0.1144 for CD8 depletion showing no statistical difference compared with not treated group, whereas P = 0.0043 for NK cell depletion showing that these cells were not needed for the therapy to work effectively). While CD4^+^ helper T cells can also be important in some cases for anti-tumor responses, it was not possible to determine their contribution in this model since anti-CD4 administration also leads to depletion of CD4^+^ regulatory T cells (Treg), which have been previously shown to play a role in responses against Renca tumors [23].

## Discussion

Previous investigations using a combination therapy of intratumoral CpG (30 µg, three times/week for 3 weeks) with 100 µg αCD137 (once weekly for 3 weeks) were performed by Smith et al [24] in BALB/c-neuT mice bearing SC TUBO tumors. The combined treatment resulted in survival of two of a total of 9 animals from two experiments, but none in a total of 10 mice from a further two experiments. In another study, SC CpG (20 µg) with IP αCD137 (100 µg) therapy (one injection of each only) was used against SC B16 melanoma in mice, with and without combination with Trp2 peptides [25]. Tumor growth with just CpG and αCD137 at this dose was less effective than when combined with Trp2 peptides.

We demonstrate in the present study that the therapy of four injections of 50 µg CpG injected IT combined with three IP injections of 100 µg αCD137 resulted in significant survival of mice injected with a variety of tumors injected SC, including B16F10 (melanoma tumor), Renca (renal carcinoma), and CT26 and MC38 (both colon cancers). However when one of these tumors, Renca, was injected orthotopically, neither injecting CpG subcutaneously or into the actual renal tumor itself, in combination with αCD137 IP, promoted survival of mice as substantial as that seen in mice injected with Renca SC. A difference between effectiveness of some therapies in SC tumors compared with orthotopic tumors has been shown previously with other mouse tumor models [26]–[30] and by us using a different immunotherapy injected IP into mice bearing orthotopic Renca tumors [11], [31]. Therefore recommendations have been made to test therapies in orthotopic models [6], [7], [29], [30], [32]–[34] which are thought to be more clinically relevant to humans. Various reasons for this difference in survival of mice with SC tumors compared with orthotopic tumors for some therapies have been surmised [27], [28], [31], [35].

Ultrasound imaging has been used in other models to treat tumor [12]–[14], but not to intratumorally treat renal tumors, to our knowledge. Several studies have used injection of orthotopic kidney tumors with therapeutic agents, but this has involved surgery on the mice to reveal the kidney and tumor and then injecting the tumor directly (but only limited to one injection timepoint) [36]–[39]. The method we have shown here demonstrates that ultrasound-guided injection of therapeutic agents is fast, minimally invasive and therefore suitable for repeated administrations and high throughput. Ultrasound imaging ease of use and sensitivity allows for a high resolution visualization of the injection of CpG medication into the tumor. This technique may be of use in other future therapies involving IT injection of kidney tumors.

We found that intratumoral injection of CpG reduced the size of primary kidney tumors compared to other treatments. In addition, we found that optimal treatment of metastatic orthotopic kidney cancers with the combined therapy of CpG plus αCD137 consisted of IV injections of CpG combined with IP injections of αCD137. This regimen statistically enhanced survival of mice, and was found to be the best route of delivery for CpG for this particular therapeutic combination. Since higher local concentrations are assumed to occur in IT delivery, this result was unexpected and future work would need to be done to explore why this is so. One potential explanation relates to the metastatic nature of the kidney tumor model, which differs from previous subcutaneous models used to compare local and systemic delivery of CpG [40], [41]. In our tumor model, Renca grows as a primary kidney tumor and spontaneously metastasizes to lung, liver and other abdominal sites. Indeed, in this model, metastasis plays a major role in disease progression and mortality. Thus, systemic delivery of CpG may have reduced the burden of metastases to a greater extent than intratumoral delivery of CpG. This is supported by the observation that intratumoral CpG-injected mice died earlier than systemic CpG-treated mice despite a reduction in primary tumor size mediated by intratumoral CpG. Systemic CpG may have impacted directly on metastases, or may have mediated its effects more efficiently than IT injection through lymphoid compartments including the lymph nodes. Previous studies have been reported in which delivery of CpG to relevant lymphoid compartments is more effective than subcutaneous injection at inducing antibody and cell-mediated responses to model antigens in a vaccine setting [42].

It is also possible that local injection of CpG into kidney tumors is less effective due to the inherent nature of the kidney tumor microenvironment, which has recently been demonstrated to be more immunosuppressive than subcutaneous tumors [31]. It may also be that different routes are appropriate for different combinations of therapy.

This study provides support for the feasibility of ultrasound-guided injection of therapeutic material into kidney tumors. However, increases in mouse survival were only of the order of several days and very few mice survived long term. This is in contrast to many previous observations using CpG and αCD137 in the subcutaneous setting where survival of a large proportion of mice was extended for several weeks, with frequent long term survivors observed [5]. The current study highlights the importance of using more physiologically relevant tumor models, involving both local and metastatic disease, when investigating therapies using immune-based agents.

## Supporting Information

Figure S1 **Setup for ultrasound-assisted intratumoral injection of kidney tumor in mice.** The ultrasound imaging equipment and screen, in relation to the injection apparatus and mouse, is shown (A). A close-up of the mouse on warm stage, with mounted needle (B). Mouse kidney tumor shown which had been injected with trypan blue to show accuracy and penetration of tumor by this method (C).
(TIF)

Figure S2 **Montage of time-lapse photography of ultrasound-assisted intratumoral injection of CpG into kidney tumor.** From left to right, the montage shows the needle tip being inserted through the skin and abdominal cavity, into the kidney tumor, and then the injection of 10 µl of CpG which can be seen as a white fluid entering the kidney tumor (most evident in the center panel).
(TIF)

Figure S3 **Individual experiments of the composite data provided in** **Figure 3** **.** In each experiment, n = 5 in the not treated groups, n = 10 for the PBS IT+αCD137 groups, n = 12–13 for the CpG IT+αCD137 groups, and n = 7–10 for the CpG IV+αCD137 groups.
(TIF)

Figure S4 **The effect of CpG IT in combined therapy on tumor growth.** The graph shows tumor size in three different experiments measured using ultrasound imaging of groups injected with CpG IT combined with αCD137 IP, compared with either no treatment on day 19 after tumor injection (A) or compared with PBS IT plus αCD137 IP (B and C) on day 17 after tumor injection.
(TIF)

Movie S1 **Ultrasound guided injection of CpG into a kidney tumor.** The abdominal cavity of a mouse is shown with the ventral skin surface uppermost. A renal tumor is evident as a relatively dense (dark) oval area within the lighter kidney. The tip of a 30-gauge needle can be seen entering the field of view from the left side of frame into the tumor. Discharge of the CpG solution and permeation of the tumor can be readily seen as a transient change in density of the tumor towards the end of the movie.
(WMV)
