# Supplementary figures and images for: Routes of Delivery for CpG and Anti-CD137 for the Treatment of Orthotopic Kidney Tumors in Mice
Source: PLoS One. 2014 May 2;9(5):e95847. doi: 10.1371/journal.pone.0095847 (PMC4008493; doi:10.1371/journal.pone.0095847)

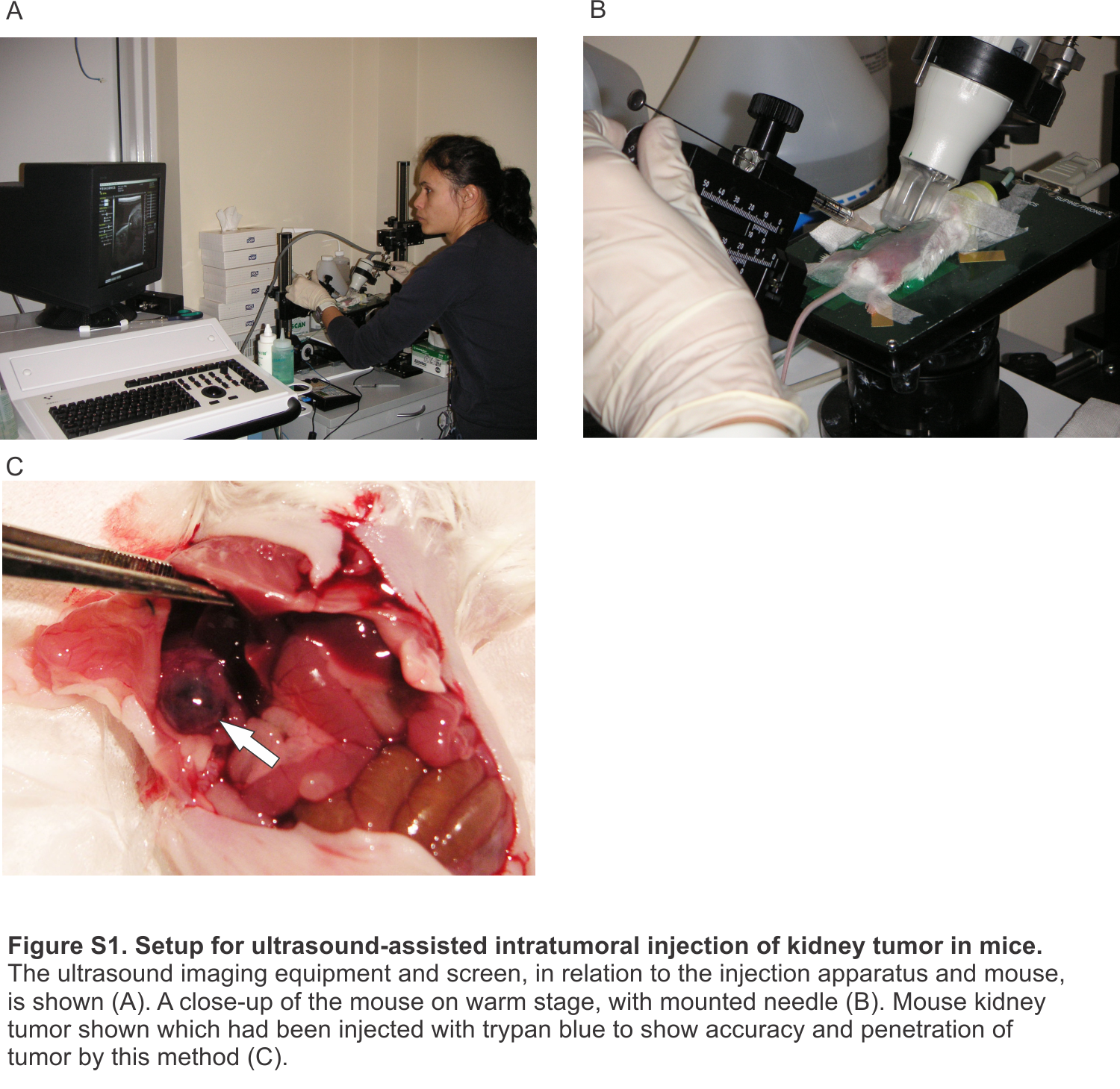

Supplement: Figure S1 — Setup for ultrasound-assisted intratumoral injection of kidney tumor in mice. The ultrasound imaging equipment and screen, in relation to the injection apparatus and mouse, is shown (A). A close-up of the mouse on warm stage, with mounted needle (B). Mouse kidney tumor shown which had been injected with trypan blue to show accuracy and penetration of tumor by this method (C). (TIF) [file pone.0095847.s001.tif]
